# Sensitive Detection of Nucleic Acids Using Subzyme Feedback Cascades

**DOI:** 10.3390/molecules25071755

**Published:** 2020-04-10

**Authors:** Nicole Hasick, Andrea Lawrence, Radhika Ramadas, Alison Todd

**Affiliations:** 1 SpeeDx, Pty Ltd., Eveleigh, NSW 2015, Australia; andreal@speedx.com.au (A.L.); radhikar@speedx.com.au (R.R.); alisont@speedx.com.au (A.T.); 2 Biotechnology and Biomolecular Sciences, The University of New South Wales, Kensington, NSW 2052, Australia

**Keywords:** catalytic DNA, deoxyribozyme, DNAzyme, PlexZyme, signal amplification, isothermal, catalytic DNA, feedback cascade, point of care

## Abstract

The development of Subzymes demonstrates how the catalytic activity of DNAzymes can be controlled for detecting nucleic acids; however, Subzymes alone lack the sensitivity required to detect low target concentrations. To improve sensitivity, we developed a feedback system using a pair of cross-catalytic Subzymes. These were individually tethered to microparticles (MP) and separated by a porous membrane rendering them unable to interact. In the presence of a target, active PlexZymes^®^ cleave a first Subzyme, which separates a first DNAzyme from its MP, allowing the DNAzyme to migrate through the membrane, where it can cleave a second Subzyme. This releases a second DNAzyme which can now migrate through the membrane and cleave more of the first Subzyme, thus initiating a cross-catalytic cascade. Activated DNAzymes can additionally cleave fluorescent substrates, generating a signal, and thereby, indicating the presence of the target. The method detected 1 fM of DNA homologous to the ompA gene of *Chlamydia trachomatis* within 30 min, demonstrating a 10,000-fold increase in sensitivity over PlexZyme detection alone. The Subzyme cascade is universal and can be triggered by any target by modifying the target sensing arms of the PlexZymes. Further, it is isothermal, protein-enzyme-free and shows great potential for rapid and affordable biomarker detection.

## 1. Introduction

Catalytic nucleic acids (CNAs) such as DNAzymes, ribozymes and PlexZymes^®^ contain the elements required for biosensing, namely, molecular recognition and transducer components. As such, they are effective tools for constructing nucleic acid biosensors. Previously, we reported the development of novel CNA structures called Subzymes [1]. Subzymes contain both a universal substrate and a CNA enzyme, and can serve as molecular intermediaries for linking target recognition with signal output. We demonstrated that the activity of certain CNAs can be inhibited by tethering Subzymes to microparticles (Subzyme-MP), and that this inhibition can be reversed by cleaving a Subzyme’s internal substrate and releasing the CNA in response to the presence of target DNA. The reactivated CNAs are capable of cleaving fluorescent-labelled reporter substrates with fast turnover rates, resulting in enhanced signal propagation, and thus providing a novel method for rapid DNA biosensing. Although Subzymes were shown to improve detection rates by ~3 fold, they currently lack the sensitivity required to detect clinically relevant concentrations of DNA [1]. In this report, we describe a strategy to further improve sensitivity by implementing a feedback cascade using a pair of cross-complementary Subzymes.

DNA-based feedback cascades can significantly enhance signal propagation for reactions where target DNA concentrations are low [2,3,4,5,6,7,8,9,10]; however, these feedback cascades have a tendency to be ‘leaky’. Any small amount of circuit instability has the potential to be amplified into a large amount of nonspecific background signal [11,12,13,14,15]. Previous studies have shown the effective use of porous membranes to control CNA reactions and minimize nonspecific molecular interactions [16,17,18]. This is exemplified in a recent study by Hong et al. (2017), whereby DNAzymes were encapsulated in selectively permeable polymethacrylic acid (PMA) capsules for metal ion sensing in living cells [16]. In this example, the PMA capsules permitted the migration of small metal ions through the capsule pores, yet restricted access to larger biomolecules such as nucleases, resulting in reduced nuclease degradation. In another strategy, DNA origami was used to encapsulate a protease enzyme so that it was unable to interact with its substrate unless released from the DNA structure upon exposure to a target [17]. Yashin et al. (2007) demonstrated the rapid diffusion of signaling molecules (DNAzyme substrates) across a porous membrane to show controlled communication between molecules without the requirement of direct contact [18]. However, although the membrane was not used to reduce the nonspecific signal, it did successfully demonstrate selective molecular exchange between reagents separated by a membrane.

Extending this concept, we have developed a cross catalytic feedback cascade that employs Porous Membrane Bags (PMBs) for selective molecular exchange. This method circumvents the problem of circuit leakage, allows sequestering of competing cross-reactions and facilitates coordinated interactions in time and space between cross-catalytic Subzyme-MPs. The protocol improves target sensitivity and specificity whilst generating results in less time. The improvements in sensitivity for nucleic acid sensing render the Subzyme cascade method suitable for some clinical diagnostic applications, and with further development, it has positive implications for future use in point of care diagnostics, particularly in developing regions.

## 2. Results and Discussion

### 2.1. Cross Catalytic Subzyme Strategy

**Figure 1:**
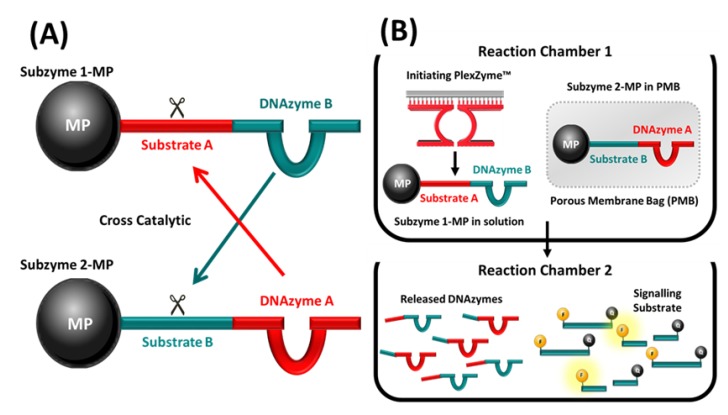
(**A**) Schema illustrating the concept of Subzyme pairs capable of cross-catalysis. Subzymes are depicted attached to microparticles (MP). Subzyme 1-MP comprises DNAzyme B linked to Substrate A which is cleavable by DNAzyme A of Subzyme 2-MP. Subzyme 2-MP comprises DNAzyme A linked to Substrate B which is cleavable by DNAzyme B of Subzyme 1-MP. (**B**) A cross-catalytic feedback cascade in which PMBs and Subzyme-MPs are used to amplify signal following detection of a DNA target. Reactions contain Subzyme 1-MP, Subzyme 2-MP inside a PMB and partzymes for the chosen DNA target. In the presence of the target, the partzymes align and form an initiating PlexZyme which cleaves Substrate A within Subzyme 1-MP separating DNAzyme B from the MP. This allows DNAzyme B to migrate through the PMB, where it can cleave Substrate B within Subzyme 2-MP, thus separating DNAzyme A from the MP. DNAzyme A is now free to migrate from the PMB into solution, where it can cleave Substrate A within Subzyme 1-MP and initiate the cascade. After a period of incubation, the supernatant containing the free DNAzymes can be transferred to another reaction chamber containing the signaling substrate. The fluorescence which is generated following the separation of the fluorophore (F) and the quencher (Q) by DNAzyme cleavage can be measured to indicate the presence or absence of target DNA.

To increase the sensitivity of the previously described Subzyme-mediated nucleic acid detection, we developed a feedback cascade using a pair of cross-complementary Subzymes. The CNAs released from one species of Subzyme-MP can serve as inputs to release and activate additional CNAs on a separate species of Subzyme-MP and so on and so forth, as depicted schematically in Figure 1A. The schema illustrates the application of a PMBs, which encloses and physically separates certain assay components from others, such that it allows the passage of certain substances through it while preventing that of others. A pair of Subzymes that contain complementary substrate and DNAzyme components are attached to microparticles (MPs) which are too large to pass though the PMB. When the Subzymes are immobilized on MPs, they remain mobile in solution; however, each will be confined within separate compartments of the assay which is defined by the PMB.

### 2.2. Feasibility of a Subzyme Feedback Cascade

**Figure 2:**
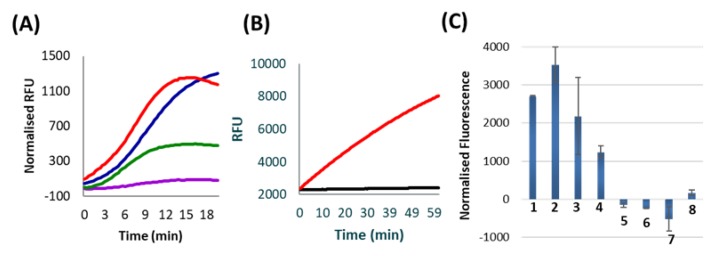
All data represent the average of two replicates. (**A**) Results from various rounds of Subzyme cross-catalysis. The product of an initial Subzyme-MP reaction was manually transferred through a series of reaction chambers containing alternating Subzyme-MPs for a total of seven rounds. The amount of active released DNAzyme was measured in separate reactions which contained labelled substrates after 1 (purple), 3 (green), 5 (blue) and 7 (red) rounds of incubation. Results are presented as normalized Relative Fluorescence Units (RFU) which is the fluorescence of no target controls (NTC) subtracted from the fluorescence of reactions containing target. (**B**) The results of an assay in which Subzyme-MPs are spatially confined within the boundaries of PMBs. Curves reflect substrate cleavage following incubation of PMB reactions containing (red line), or lacking (black line), initiating DNAzyme. Results are shown in raw RFU for 2.8 µm MP size and 0.8 µm polycarbonate PMB. (**C**) Results of a 30 min Subzyme feedback reaction using PMBs according to the schema presented in Figure 1B. Results are plotted as normalized fluorescence following a 20 min PMB incubation step and a 10 min fluorescence detection step. Reactions contain (1) *C. trachomatis* TNA (target), (2) 10 fM ompA target, (3) 2 fM ompA target, (4) 1 fM ompA target, (5) nuclease free water (NTC), (6) 1 nM PPIA off-target, (7) 1 nM p273 off-target and (8) Hu gDNA (off-target). Normalized fluorescence values were obtained for each reaction by subtracting the RFU at 0 min from the RFU at 10 min of incubation with fluorescent substrate.

The feasibility of a feedback cascade was first established by performing a multistep cross-catalytic Subzyme reaction with a complementary pair of Subzymes using the method detailed in Section 3.3. The cross-catalytic cascade, described in Section 3.2, was used to detect the target and amplify the signal in the presence of a synthetic oligonucleotide target that is homologous to a fragment of the human transferrin receptor (TFRC) gene. In this test, the active DNAzyme products released during each Subzyme reaction were sequentially transferred along a series of tubes containing alternating Subzyme-MP species. At each step, uncleaved inactive Subzyme-MPs were retained by a magnet to allow only cleaved, active DNAzymes to be transferred to the next reaction in the series. The manual transfer of supernatant was repeated for a total of seven rounds. There was an increase in the reaction rate after each round of feedback amplification, which was observed as a faster rate of fluorescent signal generated as a function of time (Figure 2A). These results demonstrated that the released DNAzymes from each round were able to cleave the substrate portion of the alternate complementary Subzymes to release additional DNAzymes in each subsequent round of amplification. An increase in the rate of signal production at each reaction round corresponds with an increased amount of DNAzyme that is accumulatively being activated, indicating that cross-catalysis has occurred. The results demonstrate proof of principle, confirming that Subzymes can function in a cross-catalytic manner.

### 2.3. Feasibility of Porous Membrane Bags (PMB)

To simplify the process outlined above so that feeedback can occur without the requirement of manual transfer between alternating Subzyme-MPs, the encapsulation of Subzyme-MPs in PMBs was explored as a mechanism of physically separating complementary Subzyme-MP pairs. The methods described in Section 3.4 were used to investigate the retention of Subzyme-MPs and the diffusion of nontethered and/or released DNAzymes through PMBs. A negligible signal was seen in the absence of initiating DNAzyme, indicating that Subzyme-MPs are unable to diffuse through the polycarbonate PMB when the diameter of the microparticle is larger than the pore size of the PMB (Figure 2B). A strong signal was observed in the presence of an initiating DNAzyme which indicates that these can diffuse into the PMB, cleave the Subzyme-MPs and release surface-bound DNAzymes. It further indicates that the released DNAzymes can diffuse out of the PMB, and when transferred together with supernatant to a new tube, the released DNAzymes can cleave fluorogenic substrates (Figure 2B). Similar results were observed for various microparticle diameters (6 µm and 8 µm), various pore diameters (0.8 µm and 2.0 µm) and using polyethersulfone PMBs (data not shown), indicating that alternative membranes can be employed for the encapsulation of Subzyme-MPs. The PMB provides a method of separating cascade components into individual compartments within a single reaction chamber whilst still permitting the exchange of their products (e.g. cleavage products). This work demonstrates that spatial separation using porous membranes is an effective mechanism for reducing or eliminating unwanted background signal in cross-catalytic reactions.

### 2.4. Subzyme Feedback Cascade Using PMBs

The method illustrated in Figure 1B was successful in detecting the *Chlamydia trachomatis* ompA gene from a sample of total nucleic acid (TNA) extracted from cultured *C. trachomatis*, as well as from a series of dilutions of oligonucleotide target homologous to ompA gene. The cross-catalytic feedback reactions could detect as little as 1 fM of *C. trachomatis* ompA gene per reaction after a 30-min total reaction time (Figure 2C). This is equivalent to 60 zeptomole or 4.2 × 10^3^ gene copies/reaction. This is a 10,000-fold improvement in sensitivity compared to the linear Subzyme reactions reported previously by our lab using the same Subzyme and PlexZyme designs, but now with an additional Subzyme to facilitate the feedback amplification. The Subzyme reactions reported previously employed only a single Subzyme-MP species and demonstrated detection of 10 pM ompA (10,000 fM) after a 60-min total reaction time [1]. Furthermore, the cross-catalytic Subzyme method also demonstrates a faster reaction time and improved sensitivity compared with other protein enzyme-free, DNAzyme-based feedback cascades reported in the literature [2,19].

In addition to increased sensitivity, the Subzyme feedback cascade targeting ompA demonstrated high specificity after a 30 min total reaction time (Figure 2C). Specificity was determined by using the Subzyme feedback to analyze a No Target Control (NTC) and several off-targets, including human genomic DNA (Hu gDNA) and oligonucleotides homologous to regions coding for the human peptidylprolyl isomerase A and plasmid p273 genes (PPIA and p273). High specificity was demonstrated by high normalized fluorescent values for samples containing *C. trachomatis* TNA, 10 fM ompA, 2 fM ompA and 1 fM ompA, and negative or low values for the NTC and off-target controls containing 1 nM PPIA, 1 nM p273 and Hu gDNA (Figure 2C).

There was minimal background signal in the presence of high concentrations of off-target templates (PPIA and p273) and Hu gDNA, indicating that the feedback cascade is specific towards the target for which it was designed (i.e. *C. trachomatis*). The detection of the *C. trachomatis* ompA gene within the TNA sample demonstrates that the feedback cascade is effective for both single stranded and double stranded DNA samples (Figure 2C).

The fluorescence generated from the two-chamber method (Figure 1B) was obtained at the end of the feedback reaction by incubating the released CNAs with fluorescent-labelled substrate. The data was not acquired in real-time, and thus, we were not able to determine a linear response to target concentration. This is illustrated when the fluorescence values from Figure 2C are compared (10 fM, 2 fM and 1 fM ompA target). The Subzyme PMB method provides a qualitative determination of the presence or absence of a target at endpoint. 

Whilst diagnosis for many infectious diseases requires the detection of pathogenic DNA/RNA concentrations as low as 10 copies/mL, there are some diseases where infectious pathogens are present at much higher concentrations and are within the scope of what the Subzyme PMB method can detect [20,21]. For example, one study reports that 85.8% (254/296) of positive *C. trachomatis* specimens had a bacterial load above the quantification limit of the qPCR (1 × 10^6^ copies/mL) [22]. Furthermore, pathogens that are known to cause urinary tract infections (UTIs) such as *Escherichia coli*, *Klebsiella spp.*, *Pseudomonas aeuroginosa* and *Staphylococcus saprophyticus* are typically present in urine samples at concentrations ranging between 1 pM–1 nM, which are also within the current detectable concentration range of the Subzyme feedback method [21,23].

Whilst the Subzyme feedback cascade shows a huge improvement in sensitivity compared to the noncascaded Subzyme reactions, concentrations lower than 1 fM of target were not detected. Additional studies are needed to improve the sensitivity to levels allowing wide application as a point of care (POC) medical diagnostic test. This includes investigations into improving the PMB construction method (discussed in Section 2.5) and exploring strategies to increase the number of active DNAzymes released from MPs for each cleavage event; for example, Subzymes could be designed to contain multiple DNAzymes.

A major advantage of this strategy is that the relative cost per reaction of the Subzyme feedback cascade is low (~$0.81 AUD/reaction). The bulk expense is attributed to the cost of the magnetic beads ($0.70 AUD/reaction) and could be reduced further by purchasing the beads in bulk or by using cheaper beads such as functionalized silica microspheres. This, combined with the simple instrumentation required for isothermal, protein-enzyme free amplification and the rapidity of the test, highlights the significant potential of the technology, particularly for use in developing nations and for development in POC devices.

### 2.5. Adaptation of the PMB Test to Detect Alternative DNA Target Sequences 

**Figure 3:**
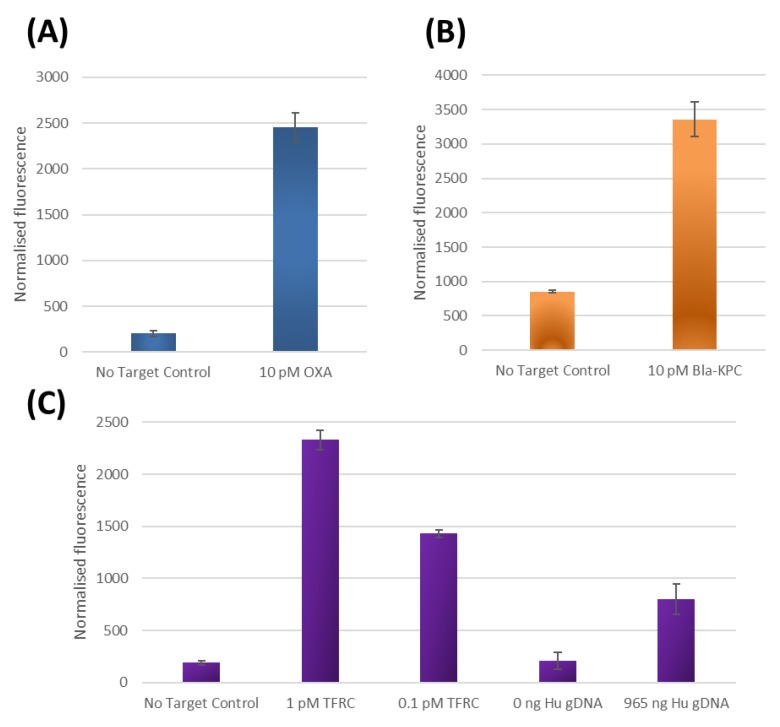
Results of three different Subzyme feedback reactions, (detailed in Figure 1B), where each reaction uses the same pair of cross-catalytic Subzymes, but each employs a different PlexZyme for detecting a different DNA target. Results are plotted as normalized fluorescence following a 20 min PMB incubation step and a 5 min fluorescence detection step by subtracting the RFU at 0 min from the RFU at 5 min. All data represent the average of two replicates. Results are shown for assays designed to detect oligonucleotide targets homologous to (**A**) bacterial OXA gene, (**B**) bacterial Bla-KPC gene and (**C**) human TFRC gene as well as detection of the TFRC gene present in human genomic DNA.

To demonstrate the universality of the Subzyme cascade, a single pair of cross-catalytic Subzymes were used in combination with three different PlexZymes for the detection of three different DNA targets. This method was successful in individually amplifying and detecting oligonucleotide targets homologous to the bacterial OXA gene, (Figure 3A), the bacterial Bla-KPC gene (Figure 3B) and the human TFRC gene (Figure 3C), as well as detecting the TFRC gene present in human genomic DNA samples (Figure 3C).

Minimal background signal was observed in the reactions lacking target compared to the fluorescence signals in the presence of OXA (Figure 3A), Bla-KPC (Figure 3B) and TFRC (Figure 3C) targets. Furthermore, the TFRC assay could also detect the TFRC gene present in extracted human gDNA samples while producing minimal background signals in the absence of DNA (Figure 3C). The results are equivalent to 25 min total reaction time and are indicative of a time required to detect 1pM (3.6 × 10^7^ gene copies) and 100 fM (3.6 × 10^6^ gene copies) of synthetic DNA and 2.8 × 10^5^ TFRC human gene copies. Since the only alteration in these experiments for the detection of different target sequence is the use of PlexZymes with target binding arms specific to either OXA, Bla-KPC and TFRC gene targets respectively, this demonstrates that the Subzyme feedback system can be universally applied for the detection of any nucleic acid target. By altering only the sensor arms of the partzymes and leaving the substrate arms unchanged, a large variety of initiating PlexZymes specific to various targets can be easily designed. The programmable and modular nature of the Subzyme feedback system eliminates the need to customize for each new target, making the assay development process faster and more straightforward.

Whilst the PMB was able to reduce the ‘leakiness’ arising from unwanted interactions between complementary Subzyme-MP species, there still remains a source of ‘leakiness’ which can sometimes be observed as low levels of nonspecific signal in the absence of target DNA (Figure 3A–C). This phenomenon became more apparent when the PMB reactions were incubated for longer than 20 min (data not shown). This is an important problem, as it restricts the possibility of extending the reaction time for detecting lower concentrations of target. This ‘leakiness’ could potentially originate from insufficient washing or alternatively, from the degradation of the Subzyme-MPs at the reaction temperature (50–55 °C). For example, if a small amount of residual free Subzyme is not completely removed by washing of Subzyme-MPs during reagent preparation, it could potentially initiate the feedback cascade, leading to high background signals and false positive outcomes. Similarly, if the Subzyme were to somehow become detached from the MP surface, i.e., by degradation or other means, it could also trigger the feedback cascade. Thus, it is important that future work aim to investigate a variety of attachment methods, attachment materials, washing procedures and storage conditions to ensure that this potential problem is appropriately addressed. It is also important to note that the PMBs were constructed manually using a hole-punch, scissors, porous membrane, oligonucleotides, microparticles and double-sided sticky tape. It is possible that contamination and human error in manufacturing the PMBs could also have played a role in the observed ‘leakiness’. Although the PMB cascade presents many exciting opportunities for DNA detection, these methods are still in their early stages and there is potential to improve the PMB manufacturing process to further improve sensitivity, ‘leakiness’ and reproducibility.

## 3. Materials and Methods

### 3.1. Subzyme Design and Attachment to Microparticles

Complementary Subzyme pairs were designed and attached to MPs according to previously published methods [1]. Subzyme 1 was designed to comprise DNAzyme B linked to Substrate A, which is cleavable by DNAzyme A of Subzyme 2. Subzyme 2 comprises DNAzyme A linked to Substrate B, which is cleavable by DNAzyme B of Subzyme 1. All oligonucleotides were synthesized and manufactured by Integrated DNA Technologies (Coralville, IA, USA). Oligonucleotide sequences are listed in Supplementary Materials A. Compel Streptavidin coated magnetic microparticles (6 µm and 8 µm) were purchased from Bangs Laboratories and Dynabeads M270 streptavidin magnetic beads (2.8 µm) were purchased from Invitrogen (Thermo Fisher Scientific, Waltham, MA, USA).

### 3.2. Fluorescence Detection Protocol

Increases in fluorescence levels, caused by the cleavage of dual-labelled substrate (Substrate-FQ) at a constant temperature of 50 °C, were measured in either FAM or Texas Red channels using a CFX96 real-time PCR detection system (Bio-Rad, Hercules, CA, USA).

### 3.3. Feasibility of Subzyme Feedback Cascade

The feasibility of a feedback cascade was first established by performing a multistep cross-catalytic Subzyme reaction using a complementary pair of Subzymes, as depicted in Figure 1A. The reaction started in chamber 1 which contained 1× PCR Buffer II (GeneAmp^®^, Life Technologies, Thermo Fisher Scientific, Waltham, MA, USA), 25 mM MgCl_2_ (Ambion, Invitrogen, Thermo Fisher Scientific, Waltham, MA, USA), 0.2 μM Partzyme A (PzA-TFRC, Supplementary Materials A), 0.2 μM Partzyme B (PzB-TFRC, Supplementary Materials A) and 13.5 μL of Subzyme 1-MP in a final volume of 250 μL. Reactions contained 100 pM of synthetic DNA Target (AF-TFRC, Supplementary Materials A) or lacked target DNA altogether (No Target Control; NTC). Chamber 1 reactions were incubated for 10 min at 50 °C. After the first incubation, a magnetic rack was used to retain uncleaved, and thus intact and inactivated, Subzyme 1-MPs and the fragments of cleaved Subzyme 1-MPs. The supernatant (containing released active DNAzymes) was transferred into a second reaction chamber. After each round of incubation with Subzyme 1-MPs (Rounds 1, 3, 5 and 7), two aliquots of the supernatant (18.5 μL) were put aside for testing. These representative portions were combined with 0.2 μM signaling substrate (Substrate 1-FQ) in a final volume of 25 μL to determine the amount of active DNAzyme that was released from the magnetic microparticles after each full round of incubation. The remaining portion of the supernatant from Round 1 (194.5 μL) was transferred into a second reaction chamber containing Subzyme 2-MPs (10.5 μL). The reactions in Chamber 2 were incubated for a further 10 min at 50 °C. Afterwards, the magnetic rack was used to transfer the supernatant (195 μL) into the third chamber whereby reactions were incubated with Subzyme 1-MPs (10 μL) for 10 min at 50 °C. Next, the supernatant (143.5 μL) was transferred into Chamber 4 (7 μL of Subzyme 2-MPs), followed by chamber 5 (6.5 μL of Subzyme 1-MPs), Chamber 6 (3.5 μL of Subzyme 2-MPs) and Chamber 7 (3 μL of Subzyme 1-MPs). In each chamber, reactions were incubated for 10 min at 50 °C. Representative aliquots of the supernatant were used to determine the amount of active DNAzyme released at the end of each round; these were measured according to the detection protocol described in Section 3.2. Texas Red fluorescence was acquired every 5 sec for a total of 200 cycles.

### 3.4. Preparation of Porous Membrane Bags (PMBs)

Porous Membrane Bags (PMBs) were constructed from inexpensive components including a porous membrane, double-sided sticky tape (X-Press It) and microparticles. Polycarbonate (0.8 µm) membrane was purchased from Sterlitech (Kent, WA, USA). Double-sided sticky tape was folded in half so that the sticky sides were folded back on themselves and the nonadhesive liner was exposed on the outer surfaces. Using a hole-punch, small wells of 6 mm diameter were punched into the folded sticky tape. After removing the liner from one side, the strip of wells punched into the sticky tape were placed onto strips of filter membrane and pressure was applied before removing the protective liner from the other side of the sticky tape. Unless otherwise specified, polycarbonate membrane (0.8 μm pore diameter) was used. An aliquot (1× final Subzyme-MP concentration) of Subzyme-MP complex was added inside the wells and left to dry at room temperature for approximately 30 s. The wells were covered with another strip of porous membrane and the PMB was trimmed into a circular disc using scissors. Finally, the PMB was placed inside a 2 mL Eppendorf tube and was only used in an experiment on the same day of preparation. All equipment, including the hole punch and scissors, were thoroughly cleaned with DNAexitusPlus™ (Bio-Strategy, Tingalpa, Australia) followed by ethanol (POCD Healthcare, North Rocks, Australia) before and after each experiment.

### 3.5. Feasibility of Porous Membrane Bags (PMB)

The following experiment was performed to determine if Subzyme-MPs are retained by PMBs made of polycarbonate material (0.8 µM). A second aim of this experiment was to determine if free DNAzymes could diffuse in and out of the PMBs and if a DNAzyme is able to cleave the substrate regions within enclosed Subzyme-MPs to release surface-bound DNAzymes. The reactions contained 1× PCR Buffer II, 45 mM MgCl_2_ and 1 PMB containing Subzyme 3-MPs in a 60 μL final volume. Reactions either contained 2 nM of initiating DNAzyme 1 (Dz1) or did not contain Dz1 as a control to determine the background signal of the enclosed Subzyme-MPs. Reactions were incubated at 50 °C for 20 min in 2 mL tubes on a heat block. After the initial incubation, the samples were transferred from the heat-block and briefly centrifuged. A 48 μL aliquot of the reaction solution was transferred to a 96-well plate (Bio-Rad) and mixed with 2 μL (0.2 μM final concentration) of signaling substrate (Substrate 2-FQ). The fluorescent signal from the Texas red channel was detected every 10 sec for a total of 150 cycles according to the isothermal protocols described in Section 3.2.

### 3.6. Subzyme Feedback Cascade Using PMBs

The cross-catalytic feedback cascade illustrated in Figure 1B was used to detect the target and amplify the signal in the presence of the C. *trachomatis* ompA gene. Reactions contained one Subzyme (Subzyme 3-MP) in solution (outside the PMB) and one Subzyme (Subzyme 4-MP) on the other side of the permeable barrier (contained within the PMB). Partzyme oligonucleotides, which are complementary to both the target being detected and to the substrate sequence within Subzyme 3-MP, were present in solution (outside the PMB). In the presence of the target, the partzyme oligonucleotides can align and form an initiating PlexZyme which can cleave the Substrate sequence (Substrate A) present within Subzyme 3-MP, leading to the release of a DNAzyme (DNAzyme B) from the MP. DNAzyme B can migrate through and into the PMB and cleave the substrate sequence (Substrate B) within Subzyme 4-MP. As a result, the surface-bound DNAzyme (DNAzyme A) is separated from the MP. The DNAzyme A is then free to migrate from inside the PMB into solution outside the PMB where it can cleave Substrate A within Subzyme 3-MP and initiate a feedback cascade reaction.

Reactions contained 5 nM of Partzyme A (PzA-ompA), 5 nM of Partzyme B (PzB-ompA), 1× PCR Buffer II, 1× of Subzyme 3-MP, 1 PMB containing 1x concentration of Subzyme 4-MP, and 45 mM MgCl_2_ in 70 µL final volume. Further, reactions contained either synthetic ompA DNA target (AF-ompA) at final concentrations of 10 fM, 2 fM and 1 fM, or 1nM of synthetic off-target DNA (AF-p273 or AF-PPIA) or 135 ng of human genomic DNA (Hu gDNA), or nuclease free water (NTC). Those reactions designed to detect the ompA gene present in *C. trachomatis* TNA samples contained 25 mM MgCl_2,_ instead of 45 mM, and *C. trachomatis* TNA (serovar D) at a final concentration of 3.5 × 10^6^ gene copies. *C. trachomatis* TNA samples were obtained in vitro using standard tissue culture techniques, as described in (Supplementary Materials B). Reactions were incubated at 50 °C for 20 min in 2 mL tubes on a heat block. After the initial incubation, the samples were transferred from the heat-block and the PMBs were removed from the tubes and discarded. Samples were briefly centrifuged, and the tubes were placed on a magnetic rack (BioRad, Hercules, CA, USA) to separate uncleaved Subzyme 3-MPs and cleaved partial substrate 3-MP fragments from DNAzymes freed into solution. A 48 µL aliquot of the supernatant was transferred to a 96-well plate (Bio-Rad, Hercules, CA, USA) and mixed with 2 µL of substrate mix containing equimolar concentrations of Substrate 4-FQ and Substrate 5-FQ (0.2 µM final concentration). FAM fluorescence was detected every 10 sec for a total of 150 cycles according to the isothermal protocols described in Section 3.2.

### 3.7. Adaptation of the PMB Test to Detect Alternative DNA Target Sequences

Here, the same pair of Subzymes were used in combination with three different PlexZymes for the detection of different DNA targets. These experiments were performed to verify that different targets could be detected using the same feedback system. The work aimed to firstly detect target and amplify the signal in the presence of oligonucleotide targets homologous to (**A**) bacterial OXA gene, (**B**) bacterial Bla-KPC gene and (**C**) human TFRC gene, as well as to detect the TFRC gene present in human genomic DNA samples.

Subzyme-MPs (Subzyme 1-MP and Subzyme 2-MP) were prepared using previously described methods [1] with the following modifications: MPs were washed three times before Subzyme immobilisation by washing two times with 100 μL of ‘Wash Buffer A’ (100 mM NaOH, 50 mM NaCl) for three mins each and one time with 100 μL of ‘Wash Buffer B’ (100 mM NaOH) for three minutes. To immobilize the Subzymes, 5 μM of Subzyme was incubated with magnetic beads for 1 h at room temperature. A series of three washes was performed in 200 μL of ‘Incubation Buffer’ at 55 °C for 5 min, followed by two washes in 200 μL of ‘Reaction Buffer’ (15 mM MgCl_2_, 1× PCR Buffer II) at 55 °C for 5 min to remove unbound Subzyme. PMBs were prepared using nitrocellulose membrane (0.45 μm, BioRad).

All reactions contained 1× PCR Buffer II, 1× of Subzyme 1-MP, 1 PMB containing 1× concentration of Subzyme 2-MP, and 15 mM MgCl_2_ in 125 µL final volume. Reactions for the detection of TFRC contained 0.2 µM PzA-TFRC, 0.2 µM PzB-TFRC and either contained hu gDNA (965 ng) or synthetic TFRC target (AF-TFRC) at final concentrations of 1 pM or 0.1 pM or no target. Reactions for the detection of OXA contained 0.2 µM PzA-OXA, 0.2 µM PzB-OXA and either contained 10 pM of synthetic OXA DNA (AF-OXA) or no DNA. Reactions for the detection of Bla-KPC contained 0.2 µM PzA-Bla-KPC, 0.2 µM PzB-Bla-KPC and either contained 10 pM of synthetic Bla-KPC DNA (AF-Bla-KPC) or no DNA. All reactions were incubated for 20 min at 50 °C and two 48 µL aliquots of the supernatant were transferred to separate wells containing equimolar concentrations of Substrate 1-FQ and Substrate 3-FQ (0.2 µM final concentration). The fluorescent signal from the Texas Red channel was measured every 5 sec for 150 cycles, according to the isothermal protocols described in Section 3.2.

## 4. Conclusions

In conclusion, the development of a cross-catalytic Subzyme reaction has improved the sensitivity and rate of target detection for Subzymes (1 fM in 30 min) compared with the noncascading Subzyme method reported previously (10,000 fM in 60 min). Porous membranes were employed to reduce competing cross-reactions by separating cross-reactive Subzymes whilst still allowing the exchange of their products to occur. This resulted in sensitive and specific feedback amplification, as demonstrated by the 10,000-fold improvement in sensitivity and minimal background signal both in the absence of the target and in the presence of nontarget controls. The universal applicability of the method was demonstrated when three different DNA targets (OXA, Bla-KPC and TFRC) were detected using a single pair of cross-catalytic Subzymes. This shows that the Subzyme feedback cascade can be easily modified to detect any target, only requiring alteration to a partzyme’s target binding arms. Further development could lead to the application of this approach in a broad range of biosensing fields, including forensic, medical, agricultural, environmental and veterinary. The simplicity and low cost of the method makes it a pertinent candidate for diagnostics in developing regions, where disease burden is high, yet resources and funding are limited.

## Supplementary Materials

Supplementary files are available online: Supplementary Materials A: Oligonucleotide Sequences; Supplementary Materials B: Tissue culture of *Chlamydia trachomatis* and extraction of Total Nucleic Acid (TNA).
