# Supplementary material for: Sensitive Detection of Nucleic Acids Using Subzyme Feedback Cascades
Source: Molecules. 2020 Apr 10;25(7):1755. doi: 10.3390/molecules25071755 (PMC7181152; doi:10.3390/molecules25071755)
Supplement: Supplementary file 1 [file molecules-25-01755-s001.pdf]

Supplementary Materials A: Oligonucleotide Sequences

Oligonucleotide sequences are listed from 5′ to 3′. UPPERCASE bases represent DNA and lowercase bases represent RNA. Underlined bases represent DNAzymes and bases in bold represent substrates. /3BioTEG/ represent Biotin functional groups conjugated to a triethylene glycol linker. FAM- indicates the location of a FAM fluorophore, A101- indicates the location of an ATTO Rho101 fluorophore and TRB- indicates the location of a Texas red fluorophore. -FQ represents the location of an Iowa Black quencher capable of absorbing fluorescence in the range of 420 – 620 nm and -RQ represents the location of an Iowa Black quencher used for absorbing fluorescence in the range of 500 – 700 nm. iFluorT indicates the location of an internal fluorescein attached to position 5 of the thymine ring and ZEN represents an internal ZEN quencher. -3P indicates a 3′ phosphate group used to inhibit degradation by exonucleases and to block extension by DNA polymerases.

ID	Oligonucleotide Sequence
**Subzyme 1**	CATGAGTGATTCCGAGCCGGTCGAAACTTCTCTACAT/iFluorT/**CACGCCTCguCTCCTCCC**/ZEN//ZEN/TTTTTTTT/3BioTEG/
**Subzyme 2**	GGAGGAGAGGCTAGCTACAACGAGAGGCGTG/iFluorT/**GTAGAGAAGTaGATCACTCATG**/ZEN//ZEN/TTTTTTTT/3BioTEG/
**Subzyme 3**	TCACTCTAGTCTCCGAGCCGGTCGAAATGCTAACGATAGTGTT**TCACGCCTCguCTCCTCCC**TTTTTTGACTAGAGTGTTTTTTT/3BioTEG/
**Subzyme 4**	GGAGGAGAGGCTAGCTACAACGAGAGGCGTGTTTTT/iFluorT/**ATCGTTAGCATgGGACTAGAGTGA**/ZEN//ZEN/TTTTTTTT/3BioTEG/
**Dz1**	GGGAGGAGAGGCTAGCTACAACGAGAGGCGTG
**AF-TFRC**	AGTCTGTTTTCCAGTCAGAGGGACAGTCTCCTTCCATATTCC
**AF-ompA**	CTTGCGATCCTTGCACCACTTGGTGTGACGCTATCAGC
**AF-Oxa**	ATTCCAGAGCACAACTACGCCGTGTGATTTATGTTCAGTAAAGTG
**AF-Bla-KPC**	CCGGTTTTGTCTCCGACTGCCCAGTCTGCCGGCACCG
**Substrate 1-FQ**	**TRB-TTGTAGAGAAGTaGATCACTCATG-RQ**
**Substrate 2-FQ**	**A101-ATCGTTAGCATgGGACTAGAGTGA-RQ**
**Substrate 3-FQ**	**A101-ATCACGCCTCguCTCCTCCCAG-RQ**
**Substrate 4-FQ**	**FAM-ATCGTTAGCATgGGACTAGAGTGA-FQ**
**Substrate 5-FQ**	**FAM-ATCACGCCTCguCTCCTCCCAG-FQ**
**PzA-TFRC**	GGAATATGGAAGGAGACTGTCACAACGAGAGACGTCGG-3P
**PzB-TFRC**	GCGGTAGAGGAGGCTAGCTCCTCTGACTGGAAAACAGACT-3P
**PzA-ompA**	CCCATACGCATGCTGATAGCACAACGAGAGGCGTGA-3P
**PzB-ompA**	GGGAGGAGAGGCTAGCTGTCACACCAAGTGGTGCAAG-3P
**PzA-Oxa**	CACTTTACTGAACATAAATCACAGACAACGAGAGGCGTGAT-3P
**PzB-Oxa**	CTGGGAGGAGAGGCTAGCTGGCGTAGTTGTGCTCTGGAAT-3P
**PzA-Bla-KPC**	CGGTGCCGGCAGACTGGGACAACGAGAGGCGTG-3P
**PzB-Bla-KPC**	GGAGGAGAGGCTAGCTCAGTCGGAGACAAAACCGG-3P

Supplementary Materials B: Tissue culture of Chlamydia trachomatis and extraction of Total Nucleic Acid (TNA)

Chlamydia trachomatis (serovar D) TNA samples were obtained in vitro, using standard tissue culture techniques as described below. The human epithelial cell line (HEp-2) (ATCC® CCL-23™, Manassas VA, USA) were grown in Dulbecco's Modified Eagle medium (DMEM) (Sigma Aldrich, St. Louis, MO, USA) supplemented with 10% heat-inactivated Fetal Bovine Serum (FBS) (Sigma Aldrich, St. Louis, MO, USA), 100 mg/mL Streptomycin (Gibco®, Invitrogen Corporation, Thermo Fisher Scientific, Waltham, MA, USA), 50 mg/mL Gentamicin (Gibco® by Life Technologies, Thermo Fisher Scientific, Waltham, MA, USA) and 20 mM Glutamine (Sigma Aldrich, St. Louis, MO, USA), incubated at 37 °C with 5% CO2. Chlamydia trachomatis was inoculated on a HEp-2 monolayer, present on a T75 flask (Nunc™, Thermo Fisher Scientific, Waltham, MA, USA) with a Multiplicity of Infection (MOI) of 1. The infection was completed by centrifugation-assisted inoculation at 500 g for 30 minutes at a temperature of 28 °C and subsequently incubated. At 4 hours post-infection (PI), DMEM was replaced with addition of 1mg/mL of Cychlohexamide (Sigma Aldrich, St. Louis, MO, USA) and again incubated at 37 °C with 5% CO2 until harvested. At an exponential growth phase, 24 hours PI, cells were manually harvested using a cell scraper and sucrosephosphate-glutamate (SPG) buffer (250mM sucrose, 10nM sodium phosphate and 5mM L-glutamate). Harvested material was stored for further processing at -80 °C. Total nucleic acid samples (TNA) were extracted using the QIAamp MinElute Virus Spin Kit (QIAgen, Hilden, Germany) following the standard protocol.
